# Beating the Odds: Finding Fulfillment and Success as an International Medical Graduate in Cardiothoracic Surgery

**DOI:** 10.1016/j.atssr.2026.05.001

**Published:** 2026-05-14

**Authors:** Andrew R. Brownlee

**Affiliations:** Division of Thoracic Surgery, Cedars-Sinai Medical Center, 8631 W 3rd St, Los Angeles, CA 91604

Invited Commentary:

As I look back on my own personal journey entering the field of cardiothoracic surgery as an international medical graduate (IMG), I can identify multiple moments where my career could have fallen short of my hopes and expectations. My current day practice without question far exceeds what I could have possibly imagined. As with so many highly sought after careers, success is the result of hard work, opportunity, excellent mentors, and of course, some luck.

The United States match rate for cardiothoracic surgery integrated and traditional fellowships in 2025 were 42% and 58%, respectively. The integrated programs saw an IMG match rate of 3.7% (2 IMGs of 54), whereas the traditional match had a rate 8.7%.^1^^,^^2^ These numbers seem depressingly low and represent not only the highly competitive nature of the specialty but also a hesitancy to match international trainees.

There are inherent/perceived risks to matching IMGs—differences in formal training, lack of knowledge of home institutions/mentors, and concerns around acquiring work visas, to name a few. Many of these concerns can be overinflated and result in outstanding applicants losing an opportunity to train in their desired specialty or at all.

For this reason, international applicants must make every effort to make their applications as competitive as possible. When discussing the match with IMGs as associate program director at my institution, I will often tell them that they need to work 30% harder, get 30% higher scores, and have 30% more research than their American colleagues. Even this does not guarantee a position, or even an interview. Personal experiences and training pathways vary widely, and each applicant or internationally trained physician has a unique story—although few are as streamlined as those of their domestically trained counterparts.

In this issue of *The Annals of Thoracic Surgery Short Reports*, Cubas and colleagues^3^ outline the “10 commandments” of pursuing a career in cardiothoracic surgery as an IMG. The article is clearly written from in-depth personal experience and should be taken as dogma for those undertaking this challenging and long road. Perhaps the most important recommendation is “persist.” Acquiring a training position takes unwavering hard work, preparation, diligence, and ultimately, being ready to seize an opportunity when it presents itself.

As education leadership, we must make a concerted effort to understand training paradigms of international applicants and differentiate between the actual and perceived barriers to training IMGs. There are many incredible applicants with boundless potential, and a blanket negative stigma could result in missing the next leader in American cardiothoracic surgery.
